# Giving Researchers a Headache – Sex and Gender Differences in Migraine

**DOI:** 10.3389/fneur.2020.549038

**Published:** 2020-10-22

**Authors:** Linda Al-Hassany, Jennifer Haas, Marco Piccininni, Tobias Kurth, Antoinette Maassen Van Den Brink, Jessica L. Rohmann

**Affiliations:** ^1^Division of Vascular Medicine and Pharmacology, Department of Internal Medicine, Erasmus MC University Medical Center, Rotterdam, Netherlands; ^2^Institute of Public Health, Charité - Universitätsmedizin Berlin, Berlin, Germany

**Keywords:** migraine, headache, aura, review (article), primary headache, sex, gender

## Abstract

Migraine is a common neurovascular disorder affecting ~15% of the general population. Ranking second in the list of years lived with disability (YLD), people living with migraine are greatly impacted by this especially burdensome primary headache disorder. In ~30% of individuals with migraine, transient neurological symptoms occur (migraine aura) that further increase migraine burden. However, migraine burden is differential with respect to sex. Though one-year prevalences in childhood are similar, starting with puberty, migraine incidence increases at a much higher rate in females than males. Thus, migraine over the life course occurs in women three to four times more often than in men. Attacks are also more severe in women, leading to greater disability and a longer recovery period. The sex disparity in migraine is believed to be partly mediated through fluctuations in ovarian steroid hormones, especially estrogen and progesterone, although the exact mechanisms are not yet completely understood. The release of the neuropeptide calcitonin gene-related peptide (CGRP), followed by activation of the trigeminovascular system, is thought to play a key role in the migraine pathophysiology. Given the burden of migraine and its disproportionate distribution, the underlying cause(s) for the observed differences between sexes in the incidence, frequency, and intensity of migraine attacks must be better understood. Relevant biological as well as behavioral differences must be taken into account. To evaluate the scope of the existing knowledge on the issue of biological sex as well as gender differences in migraine, we conducted a systematized review of the currently available research. The review seeks to harmonize existing knowledge on the topic across the domains of biological/preclinical, clinical, and population-level research, which are traditionally synthesized and interpreted in isolation. Ultimately, we identify knowledge gaps and set priorities for further interdisciplinary and informed research on sex and gender differences as well as gender-specific therapies in migraine.

## Introduction

Migraine is a common neurovascular disorder in the general population (1), estimated to affect 1.3 billion [95% uncertainty interval (UI) 1.2–1.4] people, corresponding to a global all-age point prevalence of 18.0% (2).

Migraine attack severity and frequency can vary over time and lead to different degrees of disability (3). Thus, globally, migraine accounts for 47.2 million (95% UI 30.0–68.7) years of life lived with disability (YLDs) (2). It ranks second in YLD among all causes of disability defined by the Global Burden of Disease study (GBD), and its burden is not localized to specific GBD regions but is globally distributed (4).

Migraine has a transient nature and is described by the International Classification of Headache Disorders (ICHD) as a primary headache disorder with recurrent unilateral headaches. Two major types of migraine are distinguished: migraine with aura and migraine without aura. In both types, the headache attacks last 4–72 h, are pulsating, and can be accompanied by nausea, photophobia, and/or phonophobia. Migraine with aura additionally presents with unilateral and fully reversible central nervous system symptoms (1).

The prevalence of migraine in women is higher than in men (2), and sex hormones are believed to play a key role in this discrepancy (5). After all, in females of childbearing age, migraine accounts for 11.2% of total YLDs (4). Sex hormones, especially fluctuations of estrogen and progesterone, are believed to impact the pathogenesis of migraine (6). Brain magnetic resonance imaging (MRI) studies have confirmed structural as well as functional differences between males and females with migraine (7).

Animal studies have further confirmed differences with respect to sex hormones implicated in migraine between male and female rats (8, 9), however, the majority of animal studies have been performed in male animals (5, 10). In contrast, male participants in clinical and population-based research studies on migraine are largely lacking, and differences with respect to sex and gender in people suffering from migraine have yet to be rigorously addressed (11).

Despite considerable research activity in the field of migraine over the last two decades (12), we still know little about the underlying mechanisms of the development of migraine (13).

Previous reviews have provided extensive narrative overviews of the available evidence regarding sex differences in migraine, considering both preclinical/biological as well as clinical aspects (11, 14, 15). However, a systematized search of the literature, to our knowledge, has not yet been conducted. This review aims to synthesize new evidence on sex and gender differences in migraine (published within the last 5 years). Using a systematized review approach to synthesize findings from different research fields, we attempted to provide a transparent and complete overview of recent relevant literature.

Furthermore, we aimed to identify areas of particular promise for future exploration and use interdisciplinary, translational knowledge to fill gaps in the individual subdomains of migraine research. Based on this comprehensive overview, we provide researchers with recommendations for further research in this field.

## Methods

We conducted a systematized review of the scientific literature concerning gender- and sex aspects of migraine in adults. We searched four databases: Embase, Medline (Ovid), Web-of-Science (Core Collection) and Google Scholar for relevant articles published between 01-01-2015 and 18-12-2019. Since we identified multiple earlier narrative reviews on the topic, we focused on the synthesis of newly published findings in an effort to avoid unnecessary overlap with previous reviews and to build directly upon these findings. Only scientific articles (including reviews) presenting sex- and/or gender-specific findings in original migraine research were considered for inclusion. We excluded articles limited to estimating well-described incidence and prevalence differences of migraine in men and women in various settings. We further excluded articles that were not focused on sex- or gender- specific aspects but had restricted study populations composed of only one specific sex or gender group. An exception was made for articles describing studies pertaining to women's biological health (e.g., menstruation, pregnancy and menopause), as new findings in this area are crucial to facilitate a better understanding of these sex differences.

Additionally, since this review focuses on migraine in adults, we did not include articles exclusively reporting findings on children or adolescents (aged <18 years), except for articles concerning menstruation or menarche. Moreover, studies investigating consequences and comorbidities of migraine, as well as those assessing efficacy and effectiveness of specific treatments and therapies were not included. We further restricted study eligibility to include articles available in English and as full-text manuscripts. We also excluded case reports and case series, book chapters, news articles, conference abstracts or other contributions, and dissertations.

The search strings used in the different databases included known synonyms of migraine and sex/gender terminology to maximize the sensitivity of the search. We used the following terms to conduct the literature search: “migraine,” “primary headache,” “sex characteristics,” “sex factors,” “gender identity,” “hormone,^*^” “intersex,^*^” “gender,^*^” “transgender,^*^” “hermaphrod,^*^” “female,^*^” “woman,^*^” “women,” “male,” “man,” “men,” “sex,” “sexual,” “sexes,” and “dimorphism.” The specific search strings can be found in the Supplementary.

We conducted the search on December 18th, 2019. Manuscripts were excluded during full-text screening for the following reasons: articles limited to prevalence, no gender-specific aspects, articles limited to one sex/gender or not reporting differences/similarities, averaging of effects across sex/gender groups (e.g., through matching on sex/gender), studies focused on comorbidities, testing of specific migraine treatments, no full-text accessible, text was part of a book chapter, and previously unidentified duplicates. After full-text screening, included articles were categorized into five domains including: basic science, epidemiology, clinical science, genetics and neuroimaging, for synthesis.

Contrary to previous works, we placed a particular emphasis on including (trans)gender and sex aspects - hormonal, behavioral, cultural as well as psychological traits - of migraine, which may provide important biological mechanistic insights. So far, a strong focus has been placed on sex differences, however, we believe that gender aspects might also play an important role in explaining the uneven distribution of migraine. Thus, we included both sex and gender aspects in this review. In this review, we define “sex” as referring to biological differences, concerning hormones, chromosomes, and sex organs, whereas we consider “gender” to refer to a social construct, encompassing enacted roles and behaviors, which are shaped by cultures and can change over time (16, 17). We believe both sex and gender are important determinants of health (18).

We acknowledge that modern definitions of sex and gender go beyond the binary in an effort to include individuals such as those identifying as intersex, transgender, genderfluid or genderqueer. Although such non-binary definitions are largely absent in the migraine literature, they have been captured as comprehensively as possible in this review. In our approach, we sought to identify important areas with some scientific consensus about sex and gender differences in migraine research.

## Results

**Figure 1:**
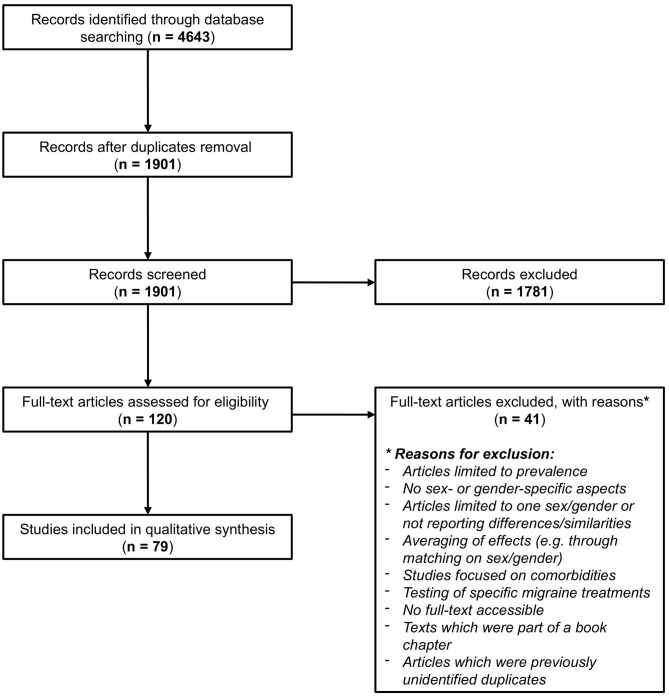
Flowchart of the review process, modified from PRISMA 2009 Flow Diagram (19).

Running the search strategy resulted in 1,901 unique retrieved articles after deduplication. Two reviewers (LAH and JH) independently screened the titles and abstracts of all retrieved articles for inclusion based on the prespecified eligibility criteria. After the first screening, there was initial agreement on inclusion of 67 articles and an additional 113 articles after discussion. For 15 articles, no consensus was found, and a third independent reviewer (JLR) was consulted to determine which of these were eligible. In total, 120 articles were eligible for full-text screening. After the second screening stage, 79 out of the 120 articles were ultimately selected for inclusion in the review. Figure 1 shows the flowchart of the review process, which was modified from PRISMA 2009 Flow Diagram (19).

### Epidemiology

In 2017, the global point prevalence of migraine was estimated to be 18.1% (95% UI 16.8–19.4), affecting ~1.3 billion (95% UI 1.2–1.4) people globally (2). Migraine is reported to be two-to-three times more prevalent in women than in men (11, 14) and have a 3- to 4-fold prevalence in women after puberty compared to men (20). This equates to a point prevalence of 22.6% (95% UI 21.1–24.3) in females and 13.5% (95% UI 12.5–14.5) in males in 2017 (2). One-year migraine attack prevalence estimates indicate active migraine in 18% of females and 6% of males globally, whereas the cumulative incidences over the lifetime are 43% and 18% respectively (11, 14, 21). The prevalence ratio between males and females does not stay constant but varies with age (11, 14, 22). The largest 1-year prevalence ratio between sexes can be observed at 30.2 years of age (11, 23).

There is a tendency of earlier onset of migraine in boys (22). The infantile migraine prevalence is similar for boys and girls, with both showing an increase beginning during puberty (11). However, this increase is steeper in women (11). According to the 2017 data, the migraine point prevalence peaks for both sexes between ages 35–39 reaching 34.5% (95% UI 29.9–39.3) and 20.3% (95% UI 17.5–27.2), respectively (2). Tonini suggests that active migraine prevalence follows a bimodal pattern with peaks around the age of 35 years and 50 years (20). However, the latest GBD data from 2017 do not show this second peak in this age group (2). In women, active migraine tends to decrease after menopausal transition (11, 14). In contrast with prevalent migraine, the peak in migraine incidence can already be observed during the age range of 20–24 years in women and 15–19 years in men (2, 11).

The global burden of migraine is indicated by the high rate of 618.4 (95% UI 392.5–898.8) years lived with disability (YLDs) per 100,000 people (2). From the latest reports in 2016, migraine ranked second among diseases leading to YLDs globally and accounted for 5.6% of YLDs globally (4). Migraine is described as particularly burdensome among middle-aged women (4). In general, migraine accounts for 6.6% (95% UI 4.6–8.7) of YLDs in women and 4.4% (95% UI 3.0–6.1) in men (2). A cross-sectional survey using a time trade-off approach found that men attribute lower health state utilities to migraine conditions compared to women (24). Especially from a life course perspective, these utilities were found to be significantly different between males and females (24). In this study, health state utilities were determined by how much of their remaining lifetime participants would be willing to sacrifice for a migraine free health state (24).

Migraine studies clearly demonstrate the impact of the disease on everyday life. A cross-sectional survey about family, relationship, career, educational, and financial aspects in migraine patients showed that overall, males and females were similarly affected in many areas (25). Gender differences were observed in that men indicated to worry more about migraine compromising their careers than women. However, migraine appeared to actually have a greater impact on women's careers compared to men's (25). Moreover, compared to men, women more often indicated they would have better overall health and less stress without migraine (25). The cross-sectional study from Brazil showed that both men and women with migraine reported more psychologically demanding jobs, less freedom in decision making in their jobs, and less social support at work (26). Especially among women, work conflicting with family and leisure was significantly associated with migraine activity (26). Furthermore, Hammond et al. found differences in associations between migraine and marital status, with men having higher odds of migraine in common-law relationships. Among older women, divorced or separated females had the highest odds of having migraine (27). Differences were also found in sexual orientation; gay and bisexual men aged 45–85 had a 50% higher odds of ever having migraine, whereas lesbian and bisexual women in the same age range had a 23% lower odds compared to heterosexual men and women, respectively (27). It was hypothesized that the underlying factor for the observed increased odds among gay and bisexual men was minority stress (27), however, to our knowledge, this has not yet been formally investigated. Also in terms of ethnicity, higher odds for migraine prevalence were observed in older non-white people compared to older white people in both sexes (27).

Conflicting results were found concerning education and income. In a recent cross-sectional study, education and social support were not associated with migraine (27). However, in another cross sectional analysis of the European Health Interview Survey for Spain, it was found that migraine was more prevalent in both men and women with lower education and income levels (28). Among older women, a social gradient was observed, with a 3% (95% CI 0.96–1.01) decrease in the odds of migraine with increasing social status, whereas in men aged 45–85 years, migraine was associated with economic status (27).

Certain kinds of physical activity were found to be associated with lower odds of having migraine, but only among older women (27). Accordingly, another study found that both females and males reporting low levels of physical activity had a higher odds of having migraine (29). Additionally, hypertension and consumption of light alcoholic beverages showed an association with migraine; however, consumption of strong alcoholic beverages was not found to be associated with migraine (29). Furthermore, associations between migraine and a history of head trauma as well as family history of headache were found in both women and men (29).

In general, migraine is characterized by a pattern of migraine attacks, remission, and relapse, with men tending to have longer remission periods than women (11, 20). Furthermore, headache attack duration is reported to be longer in women experiencing migraine attacks (20). Attack frequency and intensity seem to be similar in males and females, but severe headaches persist longer over the lifetime of women and are reported to be more bothersome (20). Symptoms including photo- and/or phonophobia, nausea, vomiting, and skin allodynia occur more often in women with migraine attacks than in men (20).

It remains unclear whether biological sex plays an important role in the transition from episodic to chronic migraine (11). In the Chronic Migraine Epidemiology and Outcomes (CaMEO) study, no major differences were observed, however, after adjusting for demographics, comorbidities, and headache features, the odds for such a transition was 43% higher for men compared to women (30).

Concerning the subtypes, migraine without aura is more common in both sexes (11). The remission rate appears unaffected by migraine subtype, with a cohort study showing that 46% were free of active migraine by the age of 50 (11, 31).

Among women, there is another differentiation of migraine subtypes based on menstruation, which distinguishes pure menstrual migraine, menstrually related migraine and non-menstrual migraine (32).

A cohort study by Scher et al. (30) found that 56.5% of males and 68.6% of females identified individual trigger factors for their migraine. While triggers including “hunger/skipping meals, thirst/dehydration, flashing/bright lights, extended screen exposure, neck strain, change in sleep patterns, caffeine [or lack of it], certain foods, [and] changes in weather/temperature” were more often reported by women, men reported common triggers to include “strong odors/scents and stress/stressful times” (30). Furthermore, in women, the time around menses, before and after onset, was often reported as a trigger factor (32).

### Basic Science and Preclinical Research

#### Sex Aspects

As the pathophysiological mechanism of migraine is complicated and involves a variety of interrelated neurological and vascular processes, we highlight the most important developments in basic research related to sex differences. It is important to realize that several aspects involved in the pathophysiology of migraine may interact with each other, and thus the topics below are tightly interrelated.

##### Central mechanisms

Previous research has shown that migraine auras might be caused by cortical spreading depression (CSD), which is a propagating wave of initial depolarization of neuronal and glial cells followed by prolonged depression (33, 34). Although not all migraine auras are followed or accompanied by headache pain (typical aura without headache), trigeminovascular activation induced by CSD may be, at least in part, responsible for the migrainous headache. Ovarian hormones have been linked to CSD susceptibility (10, 14, 15, 35, 36).

Low serotonin (5-HT) levels and reduced brain serotonin synthesis have also been linked to migraine (37). Serotonin (5-HT) is synthesized from tryptophan, which is transformed into 5-hydroxytryptophan (5-HTP) via the enzyme tryptophan hydroxylase (TPH). Estrogen is able to influence enzymes at different stages of the metabolism of tryptophan. Chauvel et al. explored the interrelations between serotonin, cortical excitability, and sex hormones in female and male rats. Their findings confirm that: (a) elevated estrogen levels increase cortical excitability, while estrogen withdrawal decreases CSD and normalizes it, (b) 5-HTP decreases the occurrence of CSD, but only in the presence of ovarian hormones and (c) in oophorectomized rats that received estradiol replacement, increased CSD was observed, which decreased after estradiol withdrawal (38).

The observed results from animal models might offer an explanation for the clinically observed association between attacks with aura and stable, high estrogen levels (e.g., during pregnancy or intake of the combined contraceptive pill). Following this line of reasoning, it seems feasible that the majority of menstrually related migraine attacks, due to estrogen withdrawal, tend to occur without aura (38).

*Familial hemiplegic migraine*. Other studies reported results from animal models of familial hemiplegic migraine type 2 (FHM2). FHM2 is a rare subtype of migraine with aura caused by mutations in the ATP1A2 gene, which encodes the α2 subunit of the Na+/K+ pump (39). This altered ion homeostasis leads to a disturbance of the sodium homeostasis (40).

An included article supported the hypothesis that estrogen plays an important role in the glutamate system in the brain (and blood). The introduction of a new α2Na+/K+-ATPase knock-in (KI) mouse model (α2^+/G301R^) showed that male mutated mice have a prolonged recovery phase after induction of CSD compared to wild type mice. Also, a link between the female sex hormone cycle and the glutamate system was established. Female α2^+/G301R^ mice showed female-specific behaviors, including hypolocomotion and reduced motor skill learning/coordination. These effects were rescued after treatment with Depoprovera, a progestin-only contraceptive, which leads to stabilized and low estrogen levels. Lastly, it was observed that the uptake function of glutamate was affected and that glutamate levels were increased in lysates from various female brain regions in mice (41).

Krost et al. also experimented with the aforementioned α2^+/G301R^ mouse model. The experiments showed increased susceptibility to CSD in α2^+/G301R^ mice; however, the results did not show sex-based effect of this increased susceptibility in adult and aged female mice compared to female mice. Nevertheless, these results suggest that this effect might be present during a restricted period of the menstrual cycle. The authors also found a decreased susceptibility to CSD after menopause in female mice but not with age in male mice, indicating an age-associated shift toward CSD (42).

We recommend a prior review by Dehghani and Karatas (43) concerning the use of FMH mouse models for more details on the study of sex differences in pathophysiological (including neurophysiological and behavioral) mechanisms involved in migraine.

*Nociception-related proteins*. Studies have demonstrated that estradiol receptors are located in trigeminal nociceptors. Therefore, the binding of estradiol might lead to the activation of extracellular signal-regulated kinase (ERK) and an increase of nociception (44).

To study the role of estrogen in the pathogenesis of migraine, Vermeer et al. utilized a multibehavioral model of migraine in rats and investigated responses to the exposure of estrogen. As compared to vehicle treatment, estradiol treatment led to a statistically significant decrease in locomotor activity as well as significant light and noise avoidance, allodynia-associated behaviors, and an enhanced acoustic startle. Moreover, estradiol treatment led to an increased expression of genes associated with estradiol signaling (expression of estrogen receptors), inflammation, vasodilation, and endogenous cannabinoid metabolism. Lastly, this treatment led to the activation of the nociception-related ERK (45).

Guo et al. investigated the role of the expression of proteins involved in the transmission of nociceptive signals, including brain-derived neurotrophic factor (BDNF) and its receptor, tropomyosin receptor kinases (TrkB), as well as ERK and its downstream target, cAMP-responsive element binding protein (CREB), in migraine by using injections of nitroglycerin to provoke migraine in rats. A positive relationship between the BDNF/TrkB and ERK/CREB pathways and the contribution of estrogen was observed. Indeed, female ovariectomized rats showed a significant decrease in the expression of BDNF, TrkB, p-CREB, and p-ERK in migraine attacks and intervals compared to rats with intact ovaries. However, the administration of estrogen recovered the expression in these ovariectomized rats. Moreover, researchers observed higher serum levels of BDNF in female than in male rats during migraine attacks (46).

##### Peripheral mechanisms

*Dysregulation of neuropeptides*. Animal studies have provided important insights into the influences of calcitonin gene-related peptide (CGRP) in migraine. This neuropeptide is thought to play a pivotal role in the pathophysiology of migraine, as it contributes to trigeminal nerve hypersensitivity and photosensitivity (47, 48). Avona et al. recently reported a female-specific response to dural administration of CGRP in a rodent migraine model assessing cutaneous periorbital hypersensitivity (49). Specifically, female rats and mice responded to lower doses of dural CGRP than their male counterparts. The authors concluded that female sex hormones have the ability to increase vasodilation in response to CGRP; however, the anatomical and physiological mechanisms explaining this difference in response have yet to be elucidated (49).

*Pro- and anti-inflammatory mediators*. Compelling evidence has been published in support of the theory that migraine is associated with a sterile inflammation of the dura, leading to increased pro-inflammatory cytokines and chemokines in plasma and cerebrospinal fluid during attacks (50–52). McIlvried et al. hypothesized that stress, being a triggering factor in migraine, induces a shift in the balance of pro- and anti-inflammatory mediator expression in dural lymphoid cells toward those that trigger a migraine attack. Moreover, they hypothesized that this effect is larger in females and that it is (partly) dependent on sympathetic postganglionic innervation of the dura. The authors tested their hypothesis in adult male and female rats. The results confirmed their hypothesis, as a sex difference was observed in (a) the increase in pro-inflammatory mediators, (b) decrease in anti-inflammatory mediators, and (c) expression of some inflammatory mediators. Moreover, (d) sympathetic postganglionic innervation only influenced the stress-induced increase of pro-inflammatory mediators in females. This article offers possibilities for different therapeutic targets in males and females (53).

We also recommend the review by Loewendrof et al. for a detailed overview of the role of mast cells, which are part of the innate immune system, and sex hormones in migraine (54).

*TRP channels*. Other structures implicated in various sensorial functions and in the pathophysiology of migraine include the non-selective cation channels Transient Receptor Potential (TRP) channels (55). Our search strategy included a review which describes the role of gonadal hormones in the activation, modulation, and regulation of the main thermoTRP channels in migraine (56).

We additionally point readers to previously published reviews from 2017 and 2018 focused on sex differences of migraine in animal models for more detailed discussions (11, 14, 15). Lastly, we recommend a review by Loewendorf et al. for a comprehensive and short summary of the literature concerning the mechanisms central sensitization, channelopathy, and sodium homeostasis in migraine (54).

#### Gender Aspects

Obviously, gender aspects in laboratory animal studies are hard to describe and might, if present at all, be mainly related to behavioral aspects. However, investigation of sex differences in animal behavior e.g., (41, 45), could be possibly interpreted as studies on gender aspects. Indeed, the way pain is experienced can be thought of as a behavioral aspect, although also sex components definitely influence pain perception. Whereas it is generally accepted in humans that pain is experienced differently in men and women, understanding the motivation behind certain behavioral traits in animals is impossible. Descriptions of behaviors that reflect pain in animal studies include avoidance responses and tending to the site of pain. It is worth mentioning that behavioral aspects in animal studies are consistent with human studies. Indeed, lower pain thresholds and an increased need for opioids have been shown in females vs. males (54).

### Translational Science: “From Bench to Bedside”

Some articles combined basic research with research in the clinical setting involving (migraine) patients, although they generally described sex differences rather than gender differences. These articles mainly aimed to describe the pathophysiology of migraine.

A study by Karkhaneh et al. determined the effect of 17β-estradiol on the expression and activity of genes involved in the process of neurogenic inflammation. The authors studied the regulation of CGRP expression, inducible nitric oxide synthase (iNOS) activity, and NO and interleukin-1beta (IL-1β) release in females with pure menstrual migraine and age-and sex-matched healthy individuals. Cultured peripheral blood mononuclear cells from these participants were treated with 17β-estradiol both, at physiological and pharmacological doses. The pharmacological dose caused a significant increase in mRNA expression of CGRP in both groups. In contrast, the physiological dose caused a significant decrease in mRNA expression of CRP, CGRP protein levels, IL-1β release, NO production and iNOS activity only in females with pure menstrual migraine (57).

In a review article, Labastida-Ramírez et al. describe sex differences of CGRP in migraine. This review highlights the profound effects varying ovarian hormones have on the trigeminovascular system in both animal and human migraine preclinical research models (58).

Ibrahimi et al. measured dermal blood flow after topical forearm application of capsaicin (the active component of chili peppers) to study endogenous release of CGRP among a group of migraine sufferers and individuals without migraine. Unlike males, females without migraine showed changes in the CGRP-dependent dermal blood flow response, which was also elevated during menstruation compared to the late-secretory phase. Females with migraine also had higher dermal blood flow responses during both menstruation and the late-secretory phase compared to females without migraine, suggesting an interrelationship between the menstrual cycle, migraine, and vascular effects mediated by CGRP (59).

Additionally, a comparable human model was developed for application of capsaicin on forehead skin, which is a trigeminal nerve–innervated dermatome, in order to study the effects of the menstrual cycle. The underlying mechanism includes activation of a TRP (TRPV1) channel by capsaicin, thereby enhancing the release of CGRP. The authors did not detect changes in trigeminovascular reactivity during the cycle in patients with menstrually related migraine in contrast to females without migraine (60).

### Clinical Science

Our search strategy retrieved a variety of original articles and reviews containing clinical evidence on the relationship between migraine, sex, and gender. As these results cover an extensive range of themes and research questions, we have classified them into different categories for the sex aspect of this review: sex hormones, reproductive events (occurring in women), biomarkers, and additional topics - which might be related to the pathophysiology of migraine. Clinical articles concerning gender aspects have been described separately for lifestyle factors, although it should be kept in mind that our two main themes - sex and gender - contain overlapping aspects.

#### Sex Aspects

##### Sex hormones

The “estrogen withdrawal hypothesis” has been expanded by several articles we included. Earlier studies consistently showed a withdrawal of estrogen in the late luteal (or premenstrual) phase, whereas the association between migraine and withdrawal of estrogen in the follicular (or periovulatory) phase has been more debated (61–64).

Pavlović et al. provide a possible explanation for the latter and show the influences of the menstrual cycle phase and timing (65). They compared the daily sex hormone levels and within-women rates of change between females with a self-reported history of migraine and controls aged 42–52 years. This study showed significant differences in the decline of urinary estrogen in the late luteal phase; individuals with migraine showed a faster decline. However, no differences in the rate of decline in the periovulatory phase and no significant differences in the peak or mean daily levels of estrogen were observed between individuals with migraine and controls. Lastly, they found that in the migraine group, the rate of decline in estrogen does not discriminate cycles with and without an acute headache. Therefore, the authors hypothesize that rapid estrogen withdrawal is not a direct trigger of migraine, but rather an endogenous characteristic and a marker for neuroendocrine vulnerability in females with migraine due to a disruption of the trigeminovascular system. Also, as progesterone has modulatory effects on estrogen in migraine, its rising levels may counteract the effects of periovulatory estrogen decline (65).

Few studies were solely performed in a male study population or attempted to investigate the role of sex hormones in this underrepresented group. It is not clear whether sex hormones modulate migraine susceptibility (risk and activity) in men as they do in women. Moreover, the role of testosterone in migraine is described in fewer studies than estrogen or progesterone.

A prospective study compared the levels and ratio of sex hormone plasma (17β-estradiol and free testosterone) in a group of medication-free, non-obese men with episodic migraine (18–74 years) to males without migraine group-matched for age and body mass index (BMI). In this study, males with migraine exhibited elevated estradiol levels, both absolute and relative to free testosterone, and showed clinical evidence of functional androgen deficiency (66). A pilot study investigated total serum testosterone levels in males aged 26–51 years with chronic migraine. Men with chronic migraine had lower total testosterone levels compared to an age-matched normative population, suggesting abnormalities in the regulation of the hypothalamus-pituitary-gonadal (HPG) axis (67). These abnormalities were then confirmed by Li et al. considering the levels of hormones. They found that levels of progesterone in males and females with migraine in the postmenopausal phase were lower compared to healthy controls. Also, they found significantly higher levels of gonadotropin-releasing hormone (GNRH) in males with migraine, in the follicular phase as well as the luteal phase in females with migraine, and in postmenopausal females with migraine compared to controls (68).

As all three studies were limited by their small sample sizes, larger studies are needed to replicate these findings and to investigate whether hormone fluctuations (as well as testosterone supplementation) might be associated with changes in migraine activity and frequency.

Lastly, supporting the hypothesis that migraine should be seen as a consequence of a loss of neurohormonal and metabolic integrity instead of a primary disorder, Dzugan et al. reported successfully treating difficult-to-treat males (*n* = 3) and females (*n* = 27) with migraine and a mean age of 46.4 years. Participants received therapy with bio-identical hormones to restore hormonal levels. As part of the restoration of the integrity between various systems (including the HPG axis) in order to manage migraine, they postulate that serum levels of all basic hormones (pregnenolone, dehydroepiandrosterone sulfate (DHEA), total testosterone, total estrogen, and progesterone) should be near to optimal with physiological cycle (69).

We direct readers to an article by Delaruelle et al. for a review covering studies investigating the influence of female and male sex hormones on primary headaches, including migraine published between 1997 and 2017 (70).

##### Reproductive milestones

Sex disparities in migraine have been also explained by the occurrence of reproductive life events, which are linked to changes in hormone levels. We sorted the included articles according to these events. Moreover, for an additional and similar overview of these sex-specific characteristics in individuals with migraine for an enhanced understanding of the impact of hormones, we recommend the reviews written by Maleki et al., Allais et al., Todd et al., Pakalnis et al. as well as the overview given by Bronet et al. (22, 71–74). We further recommend the review by Hipolito Rodrigues, which emphasizes the menopausal transition (75).

*Puberty*. Little data are available concerning risk factors for the onset of migraine in pediatric populations. Therefore, the association between pubertal timing and migraine was investigated in a recently published article. Researchers found that being a woman with an early age of menarche (early puberty) in a mainly Caucasian population was associated with a 7% lower odds of migraine at early adulthood - although pathophysiological links remain to be investigated (76).

*Menstruation*. The International Classification of Headache Disorders (ICHD) subdivides menstrual migraine (MM) (with and without aura) into pure menstrual migraine and menstrually related migraine, both with and without aura (1). Evidence regarding the association between migraine and menses has been further built upon in this review.

A relationship between self-reported migraine and menses has been reported in almost 60% of women (77). Previous studies consistently found that MM attacks have a longer duration, are more challenging to treat and are more impairing (78–82). These findings have been confirmed by a more recent study of Pavlović et al., as data from the American Migraine Prevalence and Prevention (AMPP) Study showed that self-reported migrainous headache related to menses impacted women to a larger extent. Also, women with pure menstrual migraine were more impaired due to the attacks, while women with menstrually related migraine had the overall highest burden (77). Allodynia, nausea, vomiting, and phonophobia related to the migraine attacks have been reported to be more frequent in females with MM as well (83).

The influence of hormonal changes on the development of migraine without and with aura is reflected in the results of a cross-sectional study by Taha et al. Indeed, among females with migraine, progesterone levels were significantly higher among those without aura in both phases of the menstrual cycle (follicular and luteal phase). Estradiol levels showed almost similar effects in the phases of the cycle. In the luteal phase, this hormone was significantly higher among females with migraine without aura, while in the follicular phase, it was significantly higher among females with mild migraine without aura. Moreover, a significant increase of prolactin was associated with an increase in the severity of migraine with and without aura (84). Interestingly, in the aforementioned translational study by Ibrahimi et al. (60), relatively low mean estradiol levels were detected during days 19–21 of the menstrual cycle of the patients with menstrually related migraine (60).

Conflict in the literature exists concerning pain intensity and associated symptoms as a result of the fact that these studies relied on the women's diagnosis of MM (in a clinical setting) and possibly suffered from (self-)selection bias (62, 78, 79, 81). Therefore, a prospective study by Vetvik et al. used records of headache diaries from women with and without a diagnosis of MM without aura according to the ICHD criteria. They compared the clinical characteristics of MM and non-MM without aura attacks in a random sample of women aged 30–34 years with and without MM based on diary assessment. Results of this study showed that in a representative sample of females fulfilling the ICHD criteria for MM without aura, MM without aura attacks had a significantly longer duration and were more often accompanied by severe nausea than non-MM attacks without aura. However, in women with migraine without aura who did not fulfill the ICHD criteria for MM (no diary-confirmed diagnosis of MM), no significant differences between menstrual and non-menstrual attacks were found (85).

Moreover, an exploratory study showed that chronic (15 or more days/month for more than 3 months) (1) rather than episodic migraine might be driving the associations with menstrual-cycle disorders in general and dysmenorrhea specifically (86).

Regarding other menstruation-related conditions, our search strategy retrieved two original articles about endometriosis with complementary findings. As endometriosis shares similar characteristics in terms of its clinical manifestations, epidemiology, and pathogenesis with migraine, it has been shown that migrainous headache was more frequent in the women with endometriosis than in women without endometriosis as well as in infertile women. Authors of both studies, therefore, suggest that females with migraine should be screened for endometriosis criteria and vice versa, allowing for more individualized treatment (87, 88).

*Pregnancy and breastfeeding*. The association between menstruation and migraine seems to be noticeable during pregnancy. Indeed, the large, population-based Akershus Birth Cohort (ABC) Study in Norway showed that pregnant women with self-reported MM report higher headache intensity during early pregnancy and directly postpartum compared to women without self-reported MM. Both groups showed a significant improvement during the second half of their pregnancies and directly postpartum. Moreover, the investigators report that hormonal and menstruation-related factors (premenstrual syndrome, age at menarche, and the use of hormonal contraception before pregnancy) showed no association with headache intensity, except for irregular cycles (89).

The influence of events during pregnancy, including fetal growth, on the development of migraine during adulthood has also been investigated. Data from the Norwegian population-based Nord-Trøndelag Health Study (HUNT 3) showed significant effect modification by sex in the association between being born very small for gestational age and the odds of migraine development later in life. This effect was only observed in males, probably due to differences between both sexes in adaptations of the fetal-placental unit. Altered activity of neurotransmitters and/or changes in the brain structure and connectivity might also be an explanation (90).

Several reviews have been published investigating the (beneficial) effects of pregnancy on migraine and migraine on pregnancy, as well as the influence on breastfeeding (91, 92). Moreover, Parikh describes the expected course of migraine during pregnancy and the post-partum period as well as considerations for preventive and abortive medications, although the latter topic goes beyond the scope of this review (93).

*Menopause*. Migraine shows variability in the clinical presentations in the menopausal period, as migraine attacks might improve, worsen or remain unchanged during this transition (75). The AMPP study also explored the influence of the perimenopausal status on the frequency of migraine attacks in women aged 35–65 years (94). A 1.4-fold increased risk for high-frequency headache was found during the perimenopause as compared to the premenopause. This effect might be confounded or mediated by medication overuse or depression (94). We recommend a systematic review by Ripa et al. for a more thorough overview of the articles documenting the relationship between migraine and menopause (95).

##### Additional topics

In our search, we also found a case-control study intended to build further upon the hypothesized role of iron in migraine, which plays an essential role in the synthesis of serotonin, dopamine and norepinephrine (96). Accumulation of iron in the brain, specifically in the deep brain nuclei and the periaqueductal gray matter, has been shown to be related to the migraine neurobiology - supported by earlier neuroimaging evidence (97–100). Results of this study indicated a relationship between hemoglobin, ferritin, as well as iron-deficiency anemia in subjects suffering from migraine - mainly women and girls. These findings suggest that treatment for iron-deficiency anemia or iron supplementation might be a beneficial preventive method for patients suffering from migraine coinciding with iron deficiency anemia. Indeed, both migraine and iron-deficiency (induced by heavy menstrual flow) are more common in young females (96).

Another study investigating event-related potential among individuals with migraine without aura in the interictal period showed that females had more severe abnormalities in visual neurocognitive processing under attentional conditions compared to males (101).

#### Gender Aspects

##### Migraine characteristics

Besides differences in the presentation of migraine between sexes, differences between the actual experience or manifestations of migraine have been previously described, which may be more related to gender.

A large, longitudinal, internet-based panel study showed that women reported more frequent attacks and were more likely to be disabled by their attacks than men (102). According to this cross-sectional survey, women were significantly more likely to report that the pain was unilateral and of pounding, pulsating, or throbbing in nature. Also, symptoms of nausea, photophobia, phonophobia, osmophobia, and cutaneous ictal allodynia had been reported significantly more often in women (102). Additionally, median headache duration of both migraine with and without aura seems to be longer in women than in men (103). An age-dependent variation of migraine associated symptoms (nausea, photophobia, and phonophobia) were only observed in females with migraine with and without aura, as significant changes in their frequency were mainly seen after the age of 30 (103).

##### Stress and lifestyle

A synergistic relationship between female sex and high levels of daily stress on risk of migraine headaches has been described. Females exposed to stress seem to have a higher 1-year prevalence of migraine, indicating an interplay between biological, sex-related factors and environmental stress in the progression of migraine (104).

Lifestyle factors also appear to play a role in migraine. An inverse relationship between migraine and dietary sodium intake was described by Pogoda et al. This relationship was most obvious in females with a lower BMI, while in men, the relationship did not differ by BMI after confounding adjustment (105).

### Genetics and Biomarkers

There are indications that genetic factors may explain observed phenotypic sex differences in migraine (20). In their review, Vetvik and MacGregor discuss that commonly cited genetic explanations for these differences, such as the disease being autosomal dominant in women and autosomal recessive in men, or migraine being a direct consequence of an inherited variant, might be too simplistic (11). They emphasize that migraine is polygenetic, and it is also likely that differences observed between sexes are also influenced by environmental factors.

At present, a total of 38 genomic variants have been implicated in migraine risk (106). Only one of these 38 susceptible loci is located on the X-Chromosome (11).

A link between migraine attack frequency and family history of migraine was found in males in a cross-sectional study, suggesting a certain genetic predisposition (107). Furthermore, an investigation of 12 common migraine risk loci did not find differences in risks between males or females and across different migraine subtypes (migraine with and without aura), or clinic- vs. population settings (108).

Another genotyping study, focusing specifically on the PRDM16 rs2651899 variant, found a significant difference in this variant for migraine without aura as well as the female subgroup compared to controls without migraine in all investigated models (106). The TRPM8 rs10166942 variant showed an association with migraine in male subjects and with migraine with aura, but not in females (106). However, this study had an imbalance between male and female participants and a rather small sample size (*n* = 300) for a genotyping study.

A study investigating the relationship between the progesterone receptor gene and migraine found that an PGR polymorphism was not directly connected to predisposition to migraine, however, it led to a later onset of migraine supposedly through reduced neuronal excitability in the brain (109). However, this finding was only observed in females due to the small number of male participants in the study (109).

Sazci et al. report that previous studies showed an association between methylenetetrahydrofolate reductase (MTHFR) variants C677T (rs1801133), A1298C (rs1801131) and migraine (110). Furthermore, the nicotinamide-N-methyltransferase (NNMT) variant rs694539, expressed in the brain and other nervous tissue as a cytoplasmic enzyme, was associated with migraine presence (110). The implicated MTHFR variants and the NNMT variant have been linked to higher plasma homocysteine levels, which, in turn, have been associated with migraine risk (110). However, the association between the NNMT variant and migraine was only found in women, with 4.3 times the odds for women with an AA genotype and the same magnitude of protective effect in individuals with the G allele (110).

Another genotyping study in a North Indian population (n = 500) showed a relationship between the rs10156191T variant and the odds of migraine presence in females (OR = 1.46) as well as with the odds of having migraine without aura (OR = 1.21) (111). However, due to methodological inconsistencies, the accuracy of these results is not clear. Still, these findings support previous hypotheses that diamine oxidase (DAO) increases the risk of migraine, especially in women (112). Moreover, the results suggest that the T allele in the same genetic variant has a protective effect against migraine in men (111). Both the rs2052129 and rs10156191 variants belong to the DAO gene, which is associated with high histamine levels, which, have been implicated in migraine pathophysiology (111). The presence of the investigated genetic variants is thought to lead to a reduction of DAO activity (111). Similar findings were retrieved from Caucasian Spanish (112) as well as North Indian (111) populations.

Fang et al. investigated the involvement of two specific genetic variants (rs12134493 and rs2078371), belonging to the tetraspanin 2 (TSPAN2) gene, in a Han Chinese population (113). They concluded that rs2078371 could be a potential risk factor for migraine susceptibility, especially in women and individuals with migraine without aura, which is similar to results found previously in Western populations (113). However, the biological mechanism of how TSPAN2 variation impacts migraine needs further exploration (113).

Another genotyping study (114) concentrated on twelve genetic variants previously reported to be related to migraine or the metabolism of sex hormones. A variant of rs2229741 was observed more in women with migraine compared to controls, suggesting a protective association for migraine (114). Furthermore, the GG genotype of the rs726281 variant, which is part of the estrogen receptor 1 (ESR1), lowers the susceptibility for migraine in a subgroup of women with menstrual related migraine (114). These results were retrieved from a Turkish population with a rather small sample size (*n* = 284). A meta-analysis also found that people with polymorphisms in two specific exons of the ESR1 (4 325C>G, 8 594G>A) are more susceptible to migraine, at least in a caucasian population (115).

In a small genome-wide association study, with 268 cases and 142 controls, an association between menstrual migraine and rs2506142 variant, which is located near the neuropilin 1 gene (NRP1), was found (116). The authors conclude that NRP1 might play a role in the etiology of menstrual migraine. NRP1 is involved in neuronal development processes in the nervous and vascular system, as well as in menstruation (116). It has been shown that activity of NRP1 is increased at the same time estrogen levels drop in the proliferative phase (116). Moreover, Sazci et al. report about a relation between that genetic variant in the SYNE1 and TNF genes and menstrual migraine (110).

So far, findings suggest that there might be a certain overall genetic predisposition for migraine presence. However, there are no clear differences in the association pattern between sexes. Some studies only showed significant associations of identified single genetic variants or genetic risk scores for one sex, mainly females. But due to the imbalances in sex distribution and partly small sample size of some studies, these findings should be interpreted with great caution. It is suspected that additional endogenous or exogenous factors may explain this difference (11). In the comparison between male and female migraine cases, it seems more likely that migraine has a stronger genetic basis among men than women (108). Still, Nyholt et al. report that differences in genetic risks in the subgroups are outweighed by similarities, suggesting that further investigation is needed. Thus, epigenetics came into the focus of attention, concentrating on interactions between different genetic predispositions and environmental influences (20).

There has also been research activity in the field of biomarkers, however, not very recently. In a review by Tietjen and Khubchandani (117) articles investigating the connection between various vascular biomarkers and migraine were identified (129 citations). It was reported that some studies found elevated CRP levels in female subgroups with migraine; however, the results about CRP and migraine were very inconsistent. For other biomarkers, either no sex difference was found, such as in adiponectin levels, or no sex differences were mentioned at all. If results referred to women only, the study was conducted in an exclusively female study population, thus no conclusion about sex differences could be made (117).

### Neuroimaging

The study of brain imaging contributes to an enhanced understanding of the pathophysiology of migraine, as functional, chemical, and structural differences between males and females with migraine have been observed (20, 71). Surprisingly, our search strategy only retrieved reviews, but no original articles. The review by Maleki and Androulakis explains neuroimaging patterns that distinguish females from males in migraine, also in relation to sex-related influences. Moreover, the authors mention the influence of neuroimaging in the light of some earlier described specific life events, including the perimenopause, estradiol decline and puberal phase (71). In addition, Chong et al. provide an overview of studies concerning sex difference published in the period between January 2012 and 2016 (118).

Previous functional MRI studies have shown that the posterior insular cortex and the precuneus are thicker in females compared to males with migraine (7). Also, a smaller volume of the parahippocampal gyrus was observed in these males. Moreover, female sex has been related to lower gray matter in the right dorsolateral prefrontal cortex (119).

These imaging studies also seem to explain the attribution of gender aspects in migraine, as they show gender-related differences in the involvement of regions which govern the emotional aspects of pain. Indeed, the possible involvement of the hippocampus, though not yet established, might explain gender (and sex) differences in behavioral responses to the stress and explain differences in migraine attack (20).

As highlighted by Schroeder et al. and Pavlovic et al., the majority of the neuroimaging studies have included mainly females with migraine (14, 15). Lastly, there exist very limited neuroimaging studies with considerably small sample sizes that have examined sex-related differences in migraine (71).

## Discussion

In this review, we systematically searched literature exploring sex and gender differences in migraine in the areas of epidemiology, basic science, clinical science, genetics, and neuroimaging and have summarized the findings.

Migraine is a highly prevalent and burdensome disease, particularly among women. Still, the disease is responsible for a great share of disability in both sexes. Distributional differences in gender-related lifestyle factors such as marital status, employment, and social support have also been observed among persons suffering from migraine. While many biological factors have been hypothesized to play a role in the sex difference, particularly sex hormones or sex hormonal fluctuations, a clear understanding of why migraine is different between women and men remains illusive. Many studies from which we extracted results lacked a clear operationalization of sex and gender, even when sex and/or gender aspects were the main focus of the research. Further complicating the evidence synthesis, the majority of studies presenting prevalence do not consistently use either lifetime prevalence or 1-year attack prevalence, making them difficult to compare. Other studies lack any specification of a time frame, which is indispensable for a correct interpretation of the results.

From a pathophysiological point of view, the influence of sex differences related to hormone levels on a variety of mechanisms (including the role of CGRP, cortical spreading depression, and various anti- and proinflammatory mediators) involved in migraine have primarily been studied in animals and often in isolation, as no experimental model of migraine covers all aspects of this disorder. This may be a limitation, since these processes are interrelated and are known to influence each other. Little research regarding gender roles can be conducted on a basic sciences level; however, some important behavior-related aspects have been explored. Translational studies on the topic include human studies exploring trigeminovascular reactivity in a non-invasive manner.

The majority of articles identified in this review consisted of clinical studies and reviews. Clearly, sex hormones (including estrogen, progesterone and testosterone) contribute prominently to the observed sex-related differences. Specifically, changes in the migraine phenotype (including the amount of attacks and intensity) attributable to female-specific reproductive milestones highlight the profound effects of these sex aspects, with migraine becoming more prevalent in women starting in puberty, peaking in their thirties, and steeply declining after menopause. The clinical studies indicate that gender-related aspects are also important to consider, as males and females might have different pain perceptions and experiences. Reporting bias in clinical studies should not be overlooked, as the evidence indicates males more often underreport their symptoms, which likely contributes to a continued underrepresentation in clinical (and epidemiological) studies.

In the genetics literature, specific variants have been found to be associated with certain migraine subtypes, such as migraine with aura in both sexes or menstrual migraine in women, as well as the presence of migraine in females. Certain genetic variants (rs2651899, rs694539, rs10156191, rs2078371, rs2506142) appear to make individuals more susceptible to ever having migraine. Though genetics may provide useful mechanistic insights, the individual studies acknowledge that genetic aspects alone do not provide a full explanation for the sex differences observed in this multifactorial disease. In addition, as men were underrepresented in these studies and some had small sample sizes, their conclusions should be interpreted with caution.

Surprisingly, in the field of research about biomarkers and migraine, beyond the articles included in one review from 2015 (117), we did not identify any new articles in this area. Lastly, we only found reviews and no original articles on neuroimaging describing sex- and gender related differences, highlighting another gap in the existing literature.

### Strengths and Limitations

This review has several strengths. First, unlike several narrative reviews on this topic, we developed an extensive, systematic search strategy and employed a rigorous inclusion and screening in an effort to systematically identify all recent relevant articles. We hope the thorough description of the searching steps and access to the developed search strings contribute to the transparency and reproducibility of our method. We synthesized newly published evidence in the context of the main findings of previously published review articles in an effort to provide broad topical coverage while minimizing unnecessary redundancy. Furthermore, we made an effort to go beyond biological sex aspects and also include gender aspects (behaviors, roles, etc.) where available. In an attempt to convey the distinction between both concepts, in our synthesis, we attempted to consistently operationalize sex as biological differences and gender as social construct that is shaped by cultures and varies over time. This approach stands in contrast to previous reviews and much of the original literature, in which the terms “sex” and “gender” are often used interchangeably.

Some limitations should be considered in the interpretation of our results. First, this review is limited by the articles we found through our search strategy. To limit extensive overlap with previous reviews and focus on the newest literature (2015-present), older studies were summarized more broadly based on existing reviews (11, 14, 15, 20, 22, 43, 54, 56, 58, 70–75, 91–93, 95, 117, 118). Second, we excluded articles investigating the effects of migraine therapies and treatments to focus on sex- and gender-differences in migraine as a disease, itself, rather than sex-specific differences related to individual treatment strategies, adherence, and migraine management. We acknowledge there are important (behavioral) differences between males and females with respect to treatment, but these are outside the scope of the present review (102, 120). Robbins and Bernat did not identify articles on treatment efficacy stratified by sex, illustrating that this topic also needs more attention in the field of migraine treatment research (121). Sex and gender differences in migraine comorbidities were also outside the scope of our review.

Finally, we did not provide a rigorous quality assessment of the included articles, though we made an effort to caution against over-interpretation, especially where the evidence was particularly thin. Given these aforementioned limitations, we do not claim that this review is exhaustive in terms of findings in the field of sex and gender differences in migraine, however, we believe it is quite comprehensive given its scope.

### Recommendations

Taking the above-mentioned gaps into account, we encourage further studies on sex and gender aspects in migraine across all relevant basic, clinical and population science domains and advocate for clearer operationalization of both terms - sex and gender - in future work.

Second, we observed that most studies did not contain a primary objective to identify sex- and/or gender differences. These differences were rather stated as secondary findings, implying that some studies may not have been sufficiently powered to detect differences between sex or gender strata. We advocate for *a priori* consideration of relevant research questions pertaining to sex and/or gender and investigation of sex and/or gender as a potential effect measure modifier. Furthermore, many studies matched participants with and without migraine by sex (or gender) to answer the study's primary research question, which makes inferences about the relationship between sex (or gender) and migraine impossible by design. This highlights a large methodological issue pervasive in the included literature: authors often matched for sex and provided averaged estimates, even though sex and gender differences are well-known. Therefore, a point of improvement for future studies is to avoid averaging of the effect size across of both sexes and/or genders, or to at least additionally provide stratified estimates.

Third, we echo the comments of other authors (11, 117) encouraging further research to include diverse study populations. In our review, we were explicit in our aims to identify new findings in transgender or intersex people and included these terms in the search strategy. To date, we found only two studies (122, 123) that investigated migraine in transgender people. This highlights sex and gender diversity as an insufficiently studied area in migraine research.

Fourth, basic science studies with a more representative distribution of sexes are warranted. Indeed, female animals are often underrepresented in animal studies. Although it may be justified to constrain the experimental animal population to one sex (or a specific age) for some research questions, it is a common misunderstanding that the inclusion of only male animals will lead to more reproducible results with less variation. While results of animal experiments in females may be affected by their estrous cycle and may thus be subject to intraindividual variation over time, experiments in male animals may likewise exhibit such variations for other reasons such as differences between dominant and subordinate males when housed together (124). Although most included basic science articles used a balanced distribution of males and females, there is still considerable room for improvement. Similar issues also arise in human studies, where only a few studies investigated migraine in males. As mentioned in the review by Schroeder et al. (15), societal differences and stigma surrounding this “feminized” disease should not be underestimated.

Fifth, we noticed potential for improvement in methodological quality and reporting across the included articles. In the first stage of the screening process, we were forced to exclude several articles due to methodological issues. Furthermore, in the presentation of the results, it was often difficult to discern whether the described methods were actually (and correctly) applied. Many articles reported conclusions that did not align with their stated aims, were overstated, or did not reasonably reflect their results.

Sixth, we encourage translational research of sex and gender across the three pillars - basic, clinical, and epidemiological studies - of the broad field of medicine. Although we identified existing translational research (“from benchside to bedside”), no articles assessed translational aspects from benchside to clinic or population level. Perhaps unsurprisingly, gender aspects were more often thematized in epidemiological studies, whereas sex aspects were addressed more frequently in basic science. Clinical articles showed fluidity of these domains and contained both aspects. Although the study of gender aspects in basic science might be challenging, behavioral animal studies might be an interesting area for further exploration.

Lastly, we recommend the adoption of a more holistic and interdisciplinary approach to understanding sex and gender differences in migraine across the different domains. Genetics and biomarkers alone have not provided a conclusive answer in explaining the observed sex and gender differences, and further work is needed to explore possible important intersections between genetics, pathophysiology, behavior and environment.

## Conclusion

A thorough understanding of sex and gender differences in migraine provides important insights into the pathophysiological processes involved in migraine as well as implications on a population level. Though research into these aspects in the domains of epidemiology, basic science, clinical research, genetics, and neuroimaging continues, several observed sex and gender differences remain unexplored. Therefore, future studies in migraine research should prioritize sex and gender aspects, consider using consistent definitions of these concepts, and employ suitable methods to explore these relevant differences instead of controlling for them.

## Author Contributions

LA-H and JH created the search strategy, conducted the search, screening, selection of the articles, and drafted the manuscript. TK, AM, and MP contributed with consultation on specific topics (according to their expertise) as well as synthesis of results and their interpretation. JR provided project supervision and support throughout the project by coordinating the search strategy, reviewing the articles, and drafting of the manuscript. All authors provided critical comments on the manuscript draft and approved the final version of the manuscript.

## Conflict of Interest

TK reports having contributed to an advisory board of CoLucid and a research project funded by Amgen, for which the Charité – Universitätsmedizin Berlin received an unrestricted compensation. He further reports having received honoraria from Lilly, Newsenselab, and Total for providing methodological advice, from Novartis and from Daiichi Sankyo for providing a lecture on neuroepidemiology and research methods, and from the BMJ for editorial services. AM received research grants and/or consultation fees from Amgen/Novartis, Lilly/CoLucid, Teva and Autonomic Technologies, Inc. (ATI), as well as independent support from the Dutch Research Council (NWO, Vici grant 09150181910040), the Netherlands Organization for Health Research and Development, the Dutch Heart Foundation and the Berlin Institute of Health. JR reports receiving financial support from Novartis Pharma GmbH for conducting a self-initiated research project on migraine remission in aging on a population level unrelated to this work. The remaining authors declare that the research was conducted in the absence of any commercial or financial relationships that could be construed as a potential conflict of interest.
