# Supplementary material for: Giving Researchers a Headache – Sex and Gender Differences in Migraine
Source: Front Neurol. 2020 Oct 22;11:549038. doi: 10.3389/fneur.2020.549038 (PMC7642465; doi:10.3389/fneur.2020.549038)
Supplement: Supplementary file 1 [file Table_1.pdf]

Giving Researchers a Headache - Sex and Gender Differences in Migraine
Al-Hassany L, Haas J, Piccininni M, Kurth T, MaassenVanDenBrink A and Rohmann JL (2020)

Supplementary

Search strings applied in different databases

Embase

('migraine'/exp/mj OR (migrain* OR primary-headache*):ti) AND ('sex hormone'/exp OR 'sex difference'/exp OR 'gender identity'/exp OR (hormon* OR intersex* OR gender* OR transgender* OR hermaphrod* OR female* OR woman* OR women OR male OR man OR men OR sex OR sexual OR sexes OR dimorphism*):ab,ti,kw) AND [2015-2020]/py NOT ([Conference Abstract]/lim) AND ([English]/lim)

Medline (Ovid)

(exp *"Migraine Disorders"/ OR (migrain* OR primary-headache*).ti.) AND (exp "Sex Characteristics"/ OR exp "Sex Factors"/ OR exp "Gender Identity"/ OR (hormon* OR intersex* OR gender* OR transgender* OR hermaphrod* OR female* OR woman* OR women OR male OR man OR men OR sex OR sexual OR sexes OR dimorphism*).ab,ti,kf.) NOT (news OR congres* OR abstract* OR book* OR chapter* OR dissertation abstract*).pt. AND (english).lg AND (limit 1 to yr=2015-2020)

Web-of-Science

(TI=(migrain* OR primary-headache*)) AND (TS=(hormon* OR intersex* OR gender* OR transgender* OR hermaphrod* OR female* OR woman* OR women OR male OR man OR men OR sex OR sexual OR sexes OR dimorphism*)) AND DT=(Article OR Review) AND LA=(English) AND PY=(2015-2020)

Google Scholar

intitle:migraine gender|sex|hormone|female|male
